# Surgical outcomes after reoperation for patients with recurrent presacral tumors: a retrospective study

**DOI:** 10.1186/s12957-024-03332-6

**Published:** 2024-02-15

**Authors:** Rui Li, Zhiyuan Yu, Jiahu Ye, Xin Liu, Peiyu Li, Xudong Zhao

**Affiliations:** 1 Medical School of Chinese PLA, Beijing, China; 2 Department of General Surgery, The First Medical Center, Chinese PLA General Hospital, Fuxing Road 28, Haidian District, Beijing, 100853 China; 3 School of Medicine, Nankai University, Tianjin, China; 4 Outpatient Department of Hongshankou, Jingbei Medical District, Chinese PLA General Hospital, Beijing, China

**Keywords:** Recurrent presacral tumors, Recurrence, Reoperation, Prognosis, Complications

## Abstract

**Background:**

Relevant reports on the surgical resection and prognosis of recurrent presacral tumors are limited. The objective of this study was to explore the outcomes associated with surgical resection of recurrent presacral tumors.

**Methods:**

The data of patients with recurrent presacral tumors who received surgical resection in our hospital between June 2009 and November 2018 were retrospectively analyzed.

**Results:**

Thirty-one patients, comprising 22 females and 9 males, with recurrent presacral lesions were included in our study. A posterior approach was utilized in 27 patients, an anterior approach in 1 patient, and a combined approach in 3 patients. Intraoperative complications occurred in 13 patients (41.9%), while postoperative complications occurred in 6 patients (19.4%). The length of hospital stay was significantly shorter in patients who underwent the posterior approach compared to those who underwent the anterior and combined approaches (*P* = 0.002). The operative time for the posterior approach was significantly shorter compared to both the anterior and combined approaches (*P* = 0.006). Temporary tamponade was performed for hemostasis in 4 patients, while staged resection was performed in 2 patients during the surgical treatment process. After a median follow-up period of 115.5 months, 5 patients with recurrent malignant presacral tumors succumbed to tumor recurrence after reoperation in our hospital.

**Conclusions:**

Surgical resection remains the mainstream treatment for recurrent presacral tumors. The outcomes for recurrent benign presacral tumors after surgery demonstrate favorable results, whereas further enhancements are required to improve the outcomes for recurrent malignant presacral tumors after surgery.

**Supplementary Information:**

The online version contains supplementary material available at 10.1186/s12957-024-03332-6.

## Instruction

The presacral space, also known as the retrorectal space, represents a potential anatomical region delimited by the mesorectal fascia anteriorly, the presacral fascia posteriorly, the levator ani muscle inferiorly, the peritoneal reflection superiorly, and the iliac vessels and ureters laterally [1]. Presacral tumors arise from the presacral space and exhibit a diverse histological classification, encompassing congenital, miscellaneous, neurogenic, inflammatory, and osseous subtypes [2, 3]. Presacral tumors are clinically rare entities, with an estimated prevalence of 1 in every 40,000 hospital admissions [2]. Surgical resection is the established standard treatment for presacral tumors, with reported local recurrence rates ranging from 5.0 to 20.4% following this intervention [4–10]. Previous studies showed that incomplete resection was associated with local recurrence of presacral tumors [11, 12] and decreased survival in patients with malignant presacral tumors [12]. Zhang et al. [9] observed that R1 resection was associated with recurrence for malignant presacral tumors, while secondary resections and lesion rupture were associated with recurrence for benign presacral tumors. For recurrent presacral tumors, surgical re-excision remains the cornerstone in the management, offering not only a means to excise recurrent tumors but also potential curative benefits for perineal intractable lesions associated with recurrent presacral tumors [13]. After conducting an extensive review of relevant literature, it is evident that the majority of previous studies have primarily focused on the management of primary presacral tumors, with limited information available regarding surgical resection and prognosis for recurrent presacral tumors. In this study, we present our institutional experience, outcomes and long-term follow-up results of patients with recurrent presacral tumors who underwent reoperation for recurrent lesions in our hospital through a retrospective analysis.

## Materials and methods

### Patients selection

Patients admitted for reoperation due to recurrent presacral tumors between June 2009 and November 2018 at the Department of General Surgery, Chinese People’s Liberation Army (PLA) General Hospital were included. Patients with primary presacral tumors, anal fistula, pilonidal sinus, hemorrhoids, perianal abscess, metastatic presacral tumors, and malignancy originating from the rectum or gynecological system were excluded. Patients below the age of 18 were also excluded.

### Preoperative evaluation

Reoperation in our hospital necessitated preoperative abdominal and pelvic computed tomography (CT) for all patients. When deemed necessary, magnetic resonance imaging (MRI) was required to assess the size, location, and characteristics of the presacral tumor and its relationship to the adjacent tissues. The sacral levels of the upper margin of the presacral tumors were determined preoperatively using sagittal CT or MRI scans before surgery.

### Surgical data

**Fig. 1:**
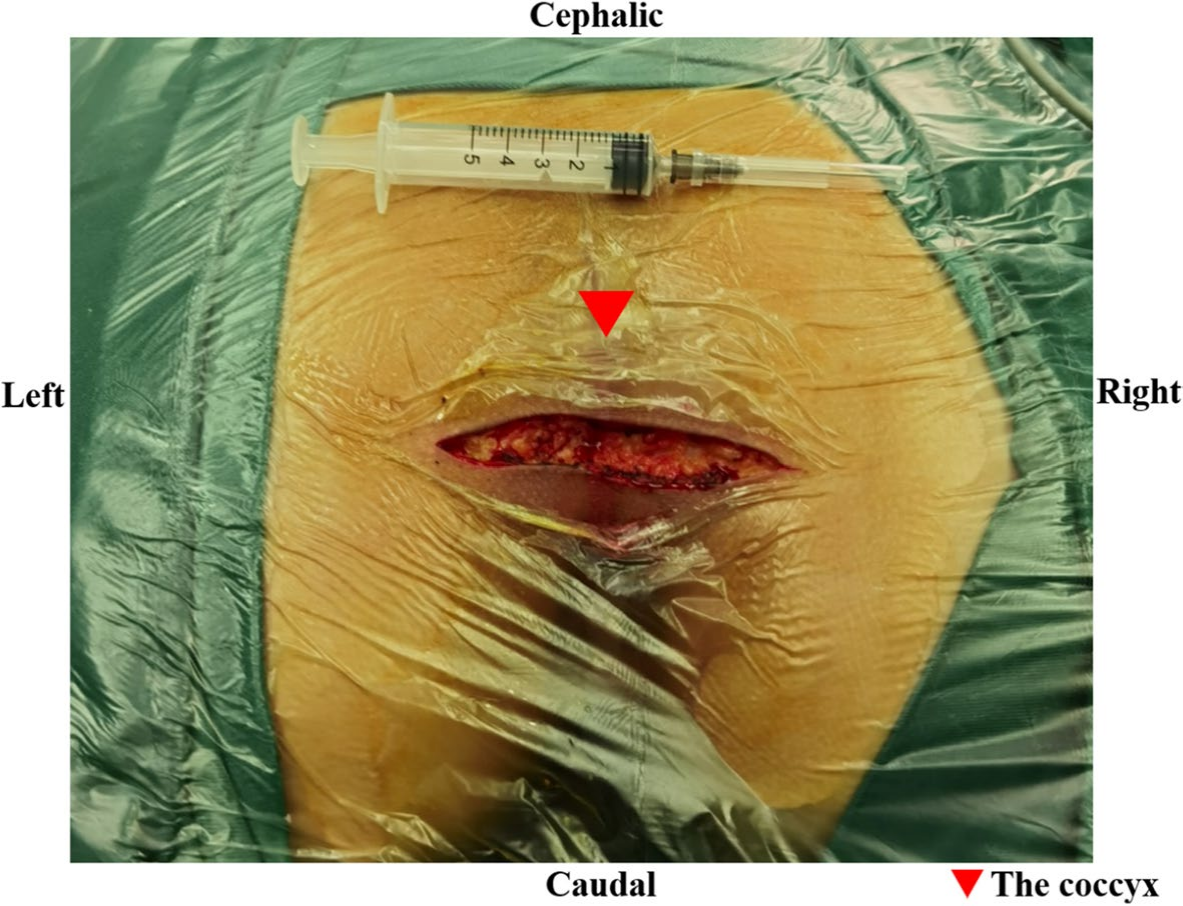
The posterior approach (a transverse incision located 1 cm below the coccyx)

**Fig. 2:**
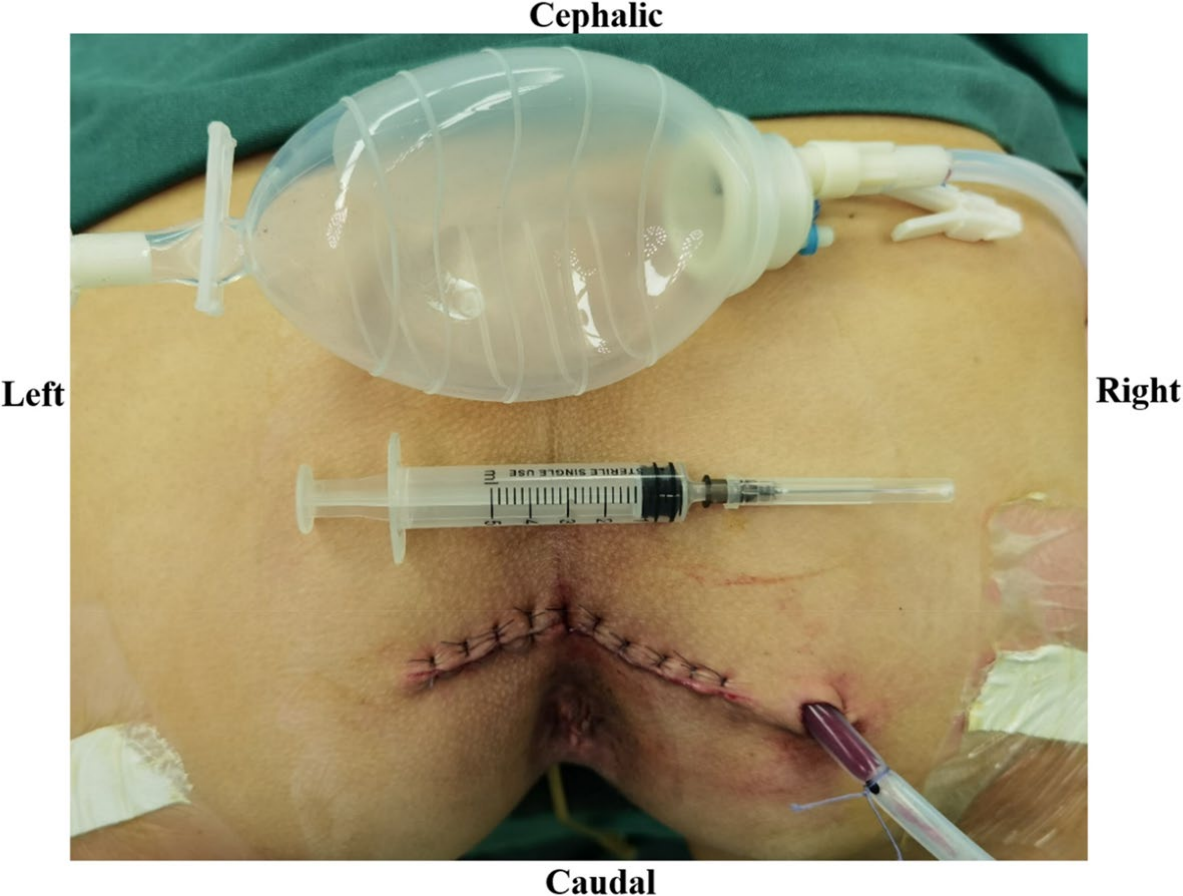
The posterior approach with a negative-pressure aspiration device

Complete resection is crucial for recurrent presacral tumors and provides patients with presacral tumors the sole opportunity for cure. Many surgical approaches have been advocated to resect presacral lesions, including the anterior approach, posterior approach, and combined approach. The anterior approach is characterized by a median incision in the lower abdomen and transabdominal tumor resection. The posterior approach is defined as a transverse incision located 1 cm below the coccyx and subsequent resection of the tumor through presacral space. (Figs. 1 and 2).

The operative time was measured from the initial incision to the final closure of the skin.

For the staged resections, the length of hospital stay was calculated by summing the durations of the two hospital stays, and the operative time was calculated by summing the durations of the two operative times.

### Intraoperative complications and postoperative morbidity

According to a multicenter study conducted in France [7], the intraoperative complications mainly encompassed tumor perforation (either intentional or unintentional), presacral bleeding, and rectal and/or bladder perforation, while postoperative 30-day morbidity predominantly comprised wound infections and pelvic abscesses. Postoperative dysuria was also included in the postoperative morbidity. The Clavien–Dindo classification was employed to assess the severity of complications [14].

### Follow-up

The follow-up was conducted through telephone interviews and outpatient visits s at specific intervals: 3, 6, 9, and 12 months after surgery in the first year. In the subsequent years (second and third), follow-up was performed every 6 months. Then, from the fourth year, follow-up was performed yearly. Pelvic CT scans were required during the follow-up period; when necessary, patients had to undergo MRI for recurrence assessment. The day of surgery initiation marked the commencement of follow-up; for patients with staged resection, the follow-up commenced immediately after the completion of the second surgical procedure. The follow-up checklist for patients with recurrent presacral tumors is shown in Supplement Table S1. The most recent follow-up time was conducted in November 2023.

### Statistical analysis

Data analysis was performed using SPSS for Windows version 20.0 (Chicago, IL, USA). Numerical data with normal distribution are expressed as the mean ± standard deviation (SD) and an independent-samples *t* test was utilized for data comparison. Numerical data without normal distribution are expressed as median (interquartile range, IQR), and the Kruskal–Wallis *H* test was utilized for data comparison. Categorical data are presented as absolute numbers and percentages. Statistical significance was achieved when *P* values were less than 0.05.

## Results

Based on the predefined inclusion and exclusion criteria, a total of 31 patients with recurrent presacral tumors who underwent surgical treatment between June 2009 and November 2018 were ultimately enrolled in this study. All patients had previously undergone surgery for presacral tumors at other institutions and presented to our hospital with recurrent presacral tumors. Among the patients, there were 22 females and 9 males, resulting in a female-to-male ratio of 2.4:1. The mean age of the enrolled patients was 36.77 ± 11.17 years (range, 20–61 years). The diameter of the presacral tumors ranged from 5 to 21 cm, with a median measurement of 11 (IQR = 7) cm. The patient demographics and clinical data have been recorded in Table 1.

**Table 1:** Demographic and clinical data of the study

Items	Number (*n* = 31)	Mean/median	*P* value
Gender			
Male	9 (29.0%)		
Female	22 (71.0%)		
Age (years)		36.77 ± 11.17	
< 37	16 (51.6%)		
≥37	15 (48.4%)		
Surgical approach			
Posterior approach	27 (87.1%)		
Anterior and combined approach	4 (12.9%)		
Tumor size (cm)		10 (IQR = 7) (range, 5 to 21)	
< 11	17 (54.8%)		
≥11	14 (45.2%)		
Length of hospital stay		14 (IQR = 11) (range, 8 to 86)	
Posterior approach	27 (87.1%)	12 (IQR = 10) (range, 8 to 45)	*P* = 0.002
Anterior and combined approach	4 (12.9%)	35 (IQR = 53.5) (range, 22 to 86)	
Pathology			
Benign	24 (77.4%)		
Intermediate	1 (3.2%)		
Malignant	6 (19.4%)		
Body mass index (BMI)			
< 18.5	2 (6.5%)		
18.5–23.9	16 (51.6%)		
24.0–27.9	9 (29.0%)		
≥28.0	4 (12.9%)		
Adjacent bone resection			
No	20 (64.5%)		
Coccygectomy	8 (25.8%)		
Coccygectomy plus partial sacrectomy	3 (9.7%)		
Co-morbidity			
No	20 (64.5%)		
Intraoperative complications	13 (41.9%)		
Postoperative complications	6 (19.4%)		
Operative time (minutes)		170 (IQR = 125) (range, 40 to 660)	
Posterior approach	27 (87.1%)	150 (IQR = 90) (range, 40 to 355)	*P* = 0.006
Anterior and combined approach	4 (12.9%)	292.5 (IQR = 205) (range, 215 to 660)	
Sacral levels of the superior margin			
L5 vertebra	1 (3.2%)		
S1 vertebra	4 (12.9%)		
S2 vertebra	4 (12.9%)		
S3 vertebra	4 (12.9%)		
Below S3 vertebra	18 (58.1%)		
Preoperative workup			
Computed tomography (CT)	31 (100%)		
Magnetic resonance imaging (MRI)	7 (22.6%)		

### Symptoms

Among a total of 31 patients, 2.3% (*n* = 10) of the patients were asymptomatic, and the rest of the patients (67.7%, *n* = 21) were symptomatic (Table 2). Among the symptomatic patients, sacral caudal pain was reported as the most prevalent complaint by 7 individuals.

**Table 2:** Symptoms observed in the study

Symptoms	Case(s) (%)
Asymptomatic	10 (32.3%)
Sacral caudal pain	7 (22.6%)
Difficult defecation	4 (12.9%)
Stomachache	3 (9.7%)
Left extremity swelling	2 (6.5%)
Changes in bowel habits	1 (3.2%)
Anal pain/discomfort	1 (3.2%)
Constipation	1 (3.2%)
Difficult urination	1 (3.2%)
Abdominal distention	1 (3.2%)

### Surgical data

Twenty-seven patients underwent surgery using the posterior approach, while 1 patient underwent surgery via the anterior approach (transabdominal excision), and 3 patients received the combined surgical approach involving both posterior and anterior approaches. During the surgical procedures, coccygectomy was performed in 8 (25.8%) patients, while coccygectomy + partial sacrectomy was performed in 3 (9.7%) patients. Complete en bloc (R0 + R1) resection was performed in 29 patients, while palliative (R2) resection was performed in 2 patients due to tumor invasion and adhesion to vital structures in the pelvis.

The operative time ranged from 40 to 660 min, with a median measurement of 170 (IQR = 125) min. The operative time for the posterior approach group ranged from 40 to 355 min, with a median measurement of 150 (IQR = 90) min, while the operative time for the anterior approach and combined approach groups ranged from 215 to 660 min, with the median measurement of 292.5 (IQR = 205) min. The operative time for the posterior approach group was significantly shorter compared to both the anterior approach and combined approach groups (*P* = 0.006).

As shown in Table 1, the median length of hospital stay for the entire patient cohort was 14 (IQR = 11) days. Specifically, patients who underwent the posterior approach had a postoperative hospital stay of 12 (IQR = 10) days, while those who underwent the anterior and combined approach had a significantly longer stay of 35 (IQR = 53.5) days (*P* = 0.002).

### Complications

The resection of a segment of the bladder wall and subsequent repair were performed in 1 female patient. Vaginal damage occurred in 1 female patient due to recurrent lesion invasion, which was subsequently repaired following the removal of the recurring tumor. The decision to perform prophylactic ileostomy in one female patient was based on the patient's compromised general condition and extensive surgical field. Tumor perforation occurred in 5 patients during surgical procedures. Uncontrolled presacral hemorrhage was observed in 4 patients, necessitating the implementation of temporary tamponade using a long piece of packing gauze to effectively manage and control the bleeding.

Staged resections were performed on two female patients, one of whom had a recurrent schwannoma and underwent her initial surgery at our hospital. However, only partial resection of the recurrent presacral tumor was conducted. Due to the superior extent of the lesion reaching L5 and the inferior extent reaching the coccyx, we performed a partial resection of approximately 50% of the tumor using a posterior approach. Considering the patient's limited tolerance for further resection, our priority was to expedite the completion of her initial surgery. After a 4-month postoperative recovery period, we performed an anterior approach to surgically resect the remaining tumor. The other female patient diagnosed with fibromatosis underwent 2 surgical procedures for resecting the recurrent lesion via a posterior approach. Due to tumor invasion into the vaginal wall and the patient’s limited surgical tolerance, only partial resection of the recurrent tumor was performed during her initial surgery. After an approximately 9-month postoperative recovery period, the remaining tumor was successfully excised during her second surgery, accompanied by resection of a portion of the vaginal wall and subsequent repair of the injured area (as shown in Table 3).

**Table 3:** Clinical data of the two patients undergoing staged resections

No	Gender and age (years)	Date of surgery	Procedures	Sacral levels of the superior margin	Bleeding (ml)	Pathology	Complications	Follow-up (months)	Recurrence
1	Male, 25	October 2013 (first); February 2014 (second)	Posterior approach (first) + anterior approach (second)	L5	300(first) + 3000 (second)	Recurrent schwannoma	No	117	No
2	Female, 25	December 2013 (first); September 2014 (second)	Posterior approach (first) + posterior approach (second)	BS3	200(first) + 200 (second)	recurrent fibromatosis	Intraoperative vaginal damage	110	No

Rectal wall damage was observed in 4 (12.9%) patients, with one case successfully treated through rectal repair and two cases requiring rectal repair + sigmoidostomy due to recurrent lesions. For the fourth rectal injury, we initially attempted the injury during surgery but were unsuccessful. Subsequently, 10 days post-surgery, a grade III rectal leakage occurred necessitating further surgical intervention. To facilitate the healing of the leakage, a transverse colostomy was performed.

Postoperative wound infection (grade I) occurred in 2 patients (6.5%), while postoperative urine retention (grade I) was observed in 1 patient (3.2%). Following conservative treatment, all patients achieved complete recovery. Another 2 patients (6.5%) experienced postoperative wound infections of grade III, necessitating surgical intervention for management.

No perioperative deaths were recorded during the study period.

### Pathology

As shown in Table 4, a total of 13 pathological types were confirmed by histological examination, including 24 (77.4%) benign lesions, 1 (3.2%) intermediate lesion, and 6 (19.4%,) malignant lesions. Among all the pathological types in our study, mature teratoma was the most prevalent benign pathological type (32.3%, *n* = 10), while liposarcoma (6.5%, *n* = 2) and teratoma with malignant transformation (6.5%, *n* = 2) were identified as the most predominant malignant pathological types.

**Table 4:** Pathologies observed in the study

Pathology	Case (s) (%)
Benign	**24 (77.4%)**
Mature teratoma	10 (32.3%)
Epidermoid cyst	5 (16.1%)
Fibromatosis	3 (9.7%)
Enterogenous cyst	2 (6.5%)
Foregut cyst	1 (3.2%)
Schwannoma	1 (3.2%)
Aggressive (invasive) angiomyxoma	1 (3.2%)
Neurofibromatosis	1 (3.2%)
Intermediate	**1 (3.2%)**
Hemangiopericytoma	1 (3.2%)
Malignant	**6 (19.4%)**
Liposarcoma	2 (6.5%)
Teratoma with malignant transformation	2 (6.5%)
Primitive neuroectodermal tumor	1 (3.2%)
Adenocarcinoma	1 (3.2%)

Complete en bloc (R0 + R1) resection was performed in 29 patients, while palliative (R2) resection was performed in 2 patients due to tumor invasion and adhesion to vital structures in the pelvis (including 1 case of recurrent liposarcoma, 1 case of recurrent fibromatosis). Following pathological confirmation, R0 resection was achieved in 25 patients, while R1 resection was performed in 4 patients, including 1 case of recurrent primitive neuroectodermal tumor (PNET), 1 case of recurrent adenocarcinoma, 1 case of recurrent teratoma with malignant transformation and 1 case of recurrent teratoma, respectively.

Among the 2 patients who underwent R2 resection, the individual with recurrent liposarcoma did not receive any adjuvant therapy post-surgery and succumbed to mortality within 6 months; the individual with recurrent fibromatosis received postoperative high-intensity focused ultrasound (HIFU) treatment after surgery at our institution. The presacral tumors remained stable, and the patient was closely monitored through imaging techniques.

Among the 4 patients who underwent R1 resection, those with recurrent teratoma exhibiting malignant transformation and recurrent PNET received postoperative chemotherapy + radiotherapy at our institution. Conversely, the patients with recurrent teratoma and recurrent adenocarcinoma did not receive any adjuvant therapy following surgery at our institution.

### Follow-up

Follow-up assessments were conducted as previously described, with the most recent follow-up occurring in November 2023. The median duration of post-surgical follow-up was 115.5 (IQR = 70, range, 6–157) months. Throughout the follow-up period, 1 patient with teratoma with malignant transformation was lost to subsequent monitoring.

During the follow-up period, tumor recurrence resulted in the death of 5 patients (16.1%). All fatalities were attributed to malignant presacral lesions, including liposarcoma (2 cases), PNET (1 case), adenocarcinoma (1 case), and teratoma with malignant transformation (1 case). These 5 patients succumbed at 6, 9, 10, 22, and 39 months post-surgery in our hospital.

One female patient with foregut cysts experienced tumor recurrence 25 months after surgery, and she declined further surgical intervention for personal reasons. Similarly, 1 male patient with teratoma experienced tumor recurrence 2 months post-surgery; however, he opted against additional surgical treatment as he remained asymptomatic. These two patients are currently being closely monitored through imaging surveillance. Among the remaining patients (*n* = 23) who underwent surgery for recurrent benign or intermediate presacral tumors at our institution, no evidence of recurrence was observed during the follow-up period.

During the long-term follow-up, constipation occurred in 2 patients, while one patient experienced chronic sacral caudal pain accompanied by constipation. Additionally, lower extremity fatigue was observed in two patients, and urinary incontinence occurred in one patient post-surgery.

## Discussion

Presacral tumors are rare clinical diseases, and recurrent presacral tumors are even more infrequent in clinical practice. Unlike primary presacral tumors, surgical resection of recurrent presacral tumors is exceptionally challenging due to surgical adhesions and alterations in the normal anatomical position. While the majority of published studies have focused on primary presacral tumors, there is a dearth of relevant research pertaining to recurrent cases. In this study, we present our surgical resection experience and long-term follow-up outcomes of recurrent presacral tumors.

According to published literature, presacral tumors exhibited a higher prevalence in females, with a female-to-male ratio of 3.7:1 in a multicentric study conducted in France [7]. Another study reported a similar female-to-male ratio of 3.5:1 [9]. The prevalence of recurrent presacral tumors also exhibited a female predominance in our study, with a female-to-male ratio of 2.4:1. The etiology behind the higher incidence of presacral tumors in female patients remains elusive. One plausible explanation is that the observed female predominance may be attributed to selection bias, as women of reproductive age are more likely to undergo digital rectal palpations compared to their male counterparts [15]. The study conducted by Li et al. [16] demonstrated that the predominance of presacral tumors in females may be attributed to fluctuations in female hormonal changes. However, further investigations are warranted to elucidate the underlying factors contributing to this female predominance in presacral tumors.

The management of patients with recurrent presacral tumors is analogous to that of patients with primary presacral tumors. Rectal palpation is recommended as the initial examination for patients suspected of recurrence in presacral masses [17], since rectal palpation can effectively detect most presacral lesions. Rectal palpation not only helps in the diagnosis of presacral tumors but also facilitates the assessment of the upper margin, which holds significance for selecting an appropriate surgical approach [15]. Subsequently, pelvic CT is essential for assessing recurrent presacral tumors. In cases where diagnostic challenges arise, an MRI examination is recommended to further evaluate the recurrence. In our study, all patients (*n* = 31) underwent preoperative pelvic CT scans and 7 patients underwent MRI scans. Preoperative CT and MRI scans can provide surgeons with valuable information, such as tumor position, size, composition (cystic or solid), and the precise spatial relationship between the presacral tumor and adjacent anatomical structures (sacrum, coccyx, rectum, blood vessels), which is crucial for surgical planning and execution. CT and MRI, known for their high sensitivity and specificity, are the two most commonly used diagnostic tools for presacral tumor diagnosis [6, 18, 19]. However, MRI exhibits superiority over CT in this regard [9].

Due to the scarcity of clinical cases, there is currently a lack of specific guidelines for selecting an appropriate surgical approach to resect presacral tumors [20]. The resection of presacral tumors can be accomplished through various surgical approaches, including the anterior approach, posterior approach, combined approach [8], laparoscopic surgery [21, 22], and robotic surgery [23, 24]. The posterior approach stands out as the most extensively employed among all the aforementioned surgical approaches [25]. The choice of surgical approach depends on the location, size, and spatial relationship of the presacral lesion with the adjacent structures [26, 27], as well as the surgical preferences of the surgeons [28]. With the development of minimally invasive surgery, laparoscopic surgery has gained increasing popularity as a viable option. Some authors [29] have attempted to resect recurrent presacral lesions using laparoscopy, yielding satisfactory short-term outcomes. However, the long-term results require further observation.

Recurrent presacral lesions pose a greater challenge for surgeons to achieve en bloc resection due to the presence of the adhesions, altered local anatomy, and inflammation resulting from previous surgeries, underscoring the paramount importance of achieving total en bloc resection during the initial surgery [30]. Due to the presence of adhesions and compression exerted by recurrent lesions on the adjacent organs and tissues, it was inevitable that damage would occur to the surrounding structures during en bloc resection. The bladder, vagina, and rectum were the most vulnerable adjacent organs. Tumor perforation usually occurs in cystic tumors, such as teratoma. Uncontrolled presacral hemorrhage was the most urgent and difficult complication in surgical procedures, requiring careful measurement by the surgeon. Furthermore, R2 resection becomes inevitable due to the patient's limited surgical tolerance or the tumor's encasement of critical pelvic tissues or structures (such as ureters and major pelvic vasculature). To reduce the recurrence rate, Li et al. [16] proposed surgical resection combined with iodine tincture treatment for some cystic presacral tumors. However, some authors [31] have expressed disagreement with this approach and question its reliability. Perhaps, in cases where only R2 resection is achieved for recurrent cystic presacral lesions, the potential efficacy of iodine tincture treatment could be explored.

Staged resections were performed in 2 female patients in our study, 1 with recurrent schwannoma and the other with recurrent fibromatosis. These 2 recurrent presacral tumors were successfully resected via staged resections. The patients were followed up for 117 months and 110 months, during which no evidence of relapse was observed. However, in our clinical practice, we consistently strive to achieve a comprehensive en-bloc resection during each surgical procedure, reserving staged resection solely for patients with pronounced debilitation and extremely limited surgical tolerance. For patients with severe debilitation, staged resection offers a viable approach to achieve complete excision of the presacral, thereby conferring enduring benefits in the long term. For patients with malignant presacral tumors, staged resection is contraindicated, as partial resection would lead to accelerated growth of the remaining malignancy and subsequently diminish the patient’s overall survival. In our patient cohort, there were only 2 patients undergoing staged resection. Further endeavors are warranted to enhance the elucidation of the efficacy of staged resection in the surgical management of recurrent presacral tumors.

Presacral vasculature injury leading to massive presacral bleeding is a challenging and potentially life-threatening intraoperative complication [32]. In our study, the packing gauze was removed after a period of 72 h, and the incision was sutured subsequently. Among these 4 patients, 1 surgical incision exhibited suboptimal healing due to preoperative radiotherapy at the corresponding site. In surgical procedures for recurrent presacral tumors, there is a higher incidence of significant presacral bleeding resulting from injury to the presacral vasculature compared to surgery for primary presacral tumors. Although temporary tamponade with a long piece of packing gauze provides a backup option to deal with bleeding, the implementation of this strategy should be exercised with prudence.

Histological type was an important factor affecting prognosis. Malignant tumors had a faster growth rate and invaded surrounding tissue more deeply. Based on the gene mutation and immune escape, malignant tumors had a higher recurrence and mortality rate [33]. In our study, 5 patients died from tumor recurrence during the follow-up period and all fatalities were attributed to malignant presacral lesions. Conversely, for recurrence in 2 patients with benign tumors, active close monitoring had become an alternative to surgery. Furthermore, the degree of radical surgery and residual tumor were the other factors affecting tumor recurrence [12]. To reduce the recurrence rate, complete en bloc (R0 + R1) resection was recommended. During the surgical procedure, the frozen section would assist the surgeon in confirming the histological type and the presence of residual tumor at the incisal margin. In cases where the primary tumor was malignant and visual assessment alone failed to ascertain the presence of residual tumor, the frozen section was indispensable.

Our current study is limited by its retrospective design and the inclusion of a small number of patients. Due to the rarity of recurrent presacral tumors in clinical practice, it is nearly impossible for a single center to conduct a prospective study. Therefore, conducting a multicenter study becomes necessary to further elucidate the long-term outcomes of these rare tumors after reoperation.

## Conclusions

Surgical resection remains the primary treatment modality for recurrent presacral tumors, with favorable outcomes observed in cases of recurrent benign presacral tumors following surgical intervention. However, there is a need to further enhance the post-surgical outcomes for recurrent malignant presacral tumors.

### Supplementary Information

**Additional file 1: Supplement Table S1.** The follow-up checklist for patients with recurrent presacral tumors after the surgical procedure.

## Data Availability

No datasets were generated or analysed during the current study.

## Abbreviations

PLA: People’s Liberation Army
CT: Computed tomography
MRI: Magnetic resonance imaging
SD: Standard deviation
IQR: Interquartile range
cm: Centimeters
HIFU: High-intensity focused ultrasound
PNET: Primitive neuroectodermal tumor
