# Supplementary material for: Surgical outcomes after reoperation for patients with recurrent presacral tumors: a retrospective study
Source: World J Surg Oncol. 2024 Feb 15;22:53. doi: 10.1186/s12957-024-03332-6 (PMC10867989; doi:10.1186/s12957-024-03332-6)
Supplement: Supplementary file 1 — Additional file 1: Supplement Table S1. The follow-up checklist for patients with recurrent presacral tumors after the surgical procedure. [file 12957_2024_3332_MOESM1_ESM.docx]

**The follow-up checklist for patients with recurrent presacral tumors**

**after the surgical procedure**

| **Items (Patient)** | **Content** |
| --- | --- |
| **Name** |  |
| **Gender** |  |
| **BMI** |  |
| **Age** |  |
| **Date of surgery** |  |
| **Date of follow-up** |  |
| **Symptoms in recent month** |  |
| **Rectal palpation** |  |
| **Blood test (Blood cell analysis)** |  |
| **Blood test (Blood biochemical examination)** |  |
| **Blood test (Tumor marker)** |  |
| **Pelvic CT** |  |
| **Pelvic MRI** |  |
| **Tumor recurrence (Yes/No)** |  |
| **Death (Yes/No)** |  |

**Annotation:**

1. **The follow-up interval:** 3, 6, 9, 12, 18, 24, 30, 36, 48, and 60 months after surgery. Subsequently, follow-up was performed yearly.
2. **Pelvic CT** scans was required during the follow-up period; when necessary, patients had to undergo **MRI** for recurrence assessment.
